# Prevalence and risk factors of methicillin-resistant *Staphylococcus aureus* colonization among HIV patients in Mekelle, Northern Ethiopia

**DOI:** 10.1186/s40064-016-2613-7

**Published:** 2016-06-24

**Authors:** Goyitom Gebremedhn, Tewelde Tesfay Gebremariam, Araya Gebreyesus Wasihun, Tsehaye Asmelash Dejene, Muthupandian Saravanan

**Affiliations:** 1 Department of Medical Microbiology and Immunology, Institute of Biomedical Science, College of Health Sciences, Mekelle University, 1871 Mekelle, Ethiopia; 2 Tigray Regional State Health Bureau, Mekelle, Ethiopia

**Keywords:** *S. aureus*, MRSA, Prevalence, HIV patients, Risk factors, CD_4_ count

## Abstract

HIV-positive individuals are at higher risk of Methicillin Resistant *Staphylococcus aureus* (MRSA) colonization and its related infection. There is limited data in the nation on the prevalence and risk factors of MRSA colonization among HIV patients. The aim of this study was to address the existing knowledge gap. Cross sectional study was carried out from September 2014 to February 2015 in three selected health centers and one general hospital. A standardized questionnaire was developed for collection of socio-demographic and clinical data. A total of 498 Nasal and throat swabs (two for each patient) were collected from 249 patients, transported and processed using standard bacteriological procedures. Data was analyzed using Chi square (X^2^) test and associated risk factors were determined. *P* < 0.05 was considered statistically significant. Out of 249 study participants, *S. aureus* was isolated from 81 (32.5 %) patients, with MRSA colonization rate of 6 (2.4 %). MRSA isolates were resistant to Ciprofloxacin and trimethoprim-sulphamethoxazole (16.7 % each), clindamycin (33.3 %) and erythromycin (50 %). However, all MRSA isolates were 100 % sensitive to Amikacin. History of hospitalization, percutaneous device usage, patients with a household member’s hospitalization and low CD_4_ count (<200 cells/mm^3^) were significantly associated with *S. aureus* colonization (p < 0.05).

## Background

Methicillin resistant *Staphylococcus aureus* (MRSA), causes severe and fatal infections such as bloodstream infections, infective endocarditis, pneumonia, skin and soft tissue infections and is a global health issue (National Clinical Effectiveness Committee 2013). Though MRSA can infect all patients, HIV infected patients are more susceptible to MRSA due to their compromised immune system (Hidron et al. 2010; Lee et al. 2005; Bozzett et al. 2001).

The anterior nares are the most consistent site for MRSA colonization (Otto 2012; Wertheim et al. 2005), yet it can also colonize the axilla, inguinal region, throat, oropharynx, wounds and gastrointestinal tract (Otto 2012; Eveillard et al. 2006; Shahin et al. 1999).

MRSA transmission is mainly by direct contact through transient carriage from the hands of health care workers, contaminated equipment or with contaminated environmental surfaces (Lowy 1998).

MRSA infections have become increasingly problematic in both health care and community settings, leading to a greater morbidity, mortality, longer hospital stays, prolonged antibiotic administration and increased treatment costs (Government of Western Australia Department of Health 2013; Wertheim et al. 2005; Filice et al. 2010; Edem et al. 2013). Existing literatures show that, HIV patients are six to 18-fold more susceptible to MRSA than the general population, and is the main cause of bacteremia and endocarditis in these patients (Popovich et al. 2010; Tumbarello et al. 2002; Burkey et al. 2008; Furuno et al. 2011; Franzetti 2006).

There are few studies on MRSA colonization in HIV negative individuals in Ethiopia (Shibabaw et al. 2013; Kejela and Bacha 2013). However, there are no data on the prevalence and risk factors for MRSA colonization among HIV patients in the country. Therefore, this study was conducted to fill the existing knowledge gap by determining the nasal and throat colonization of MRSA and related risk factors in HIV-infected persons.

## Methods

### Study design, study period and study area

A cross-sectional study was conducted from September 2014 to February 2015 in three selected health centers and one hospital giving HIV care services, namely Kasech Health Center, Mekelle Health Center, Semen Health Center and Mekelle Hospital. These health facilities were (as the report on September 2014) providing HIV care services for a total of 5989 HIV-positive patients.

#### Sample size

It was determined by taking the prevalence of MRSA from South Africa, which was 21 % (Heysell et al. 2011) using the following formula: ${\text{n }} = {\text{Z}}^{ 2} \alpha / 2 {\text{ P }}\left( { 1- {\text{P}}} \right),( 1. 9 6)^{ 2} *0. 2 1\left( { 1- 0. 2 1} \right) = { 249}$.

$${\text{d}}^{ 2} \left( {0.0 5} \right)^{ 2}$$

A proportionate allocation formula (Pandey and Verma 2008) was used to determine the sample size from each health facility.

$${\text{n}}_{\text{j}} = \frac{\text{n}}{\text{N}}{\text{ N}}_{\text{j}} \quad\,\,\, {\text{j}} = { 1},{ 2},{ 3},{\text{ k}}$$

where, k is the number of strata and n_j_ is sample size of the jth stratum

N_j_ is population size of the jth stratum

n = n_1_ + n_2_ + … + n_k_ is the total sample size

N = N_1_ + N_2_ + … + N_k_ is the total population size

The sample size of each health facility is calculated as:

n1 (Mekelle Hospital) = 160; n2 (Kasech Health center) = 28; n3 (Semen Heath Center) = 25 and n4 (Mekelle Health Center) = 43.

### Data sources and data collection

A well structured questionnaire in combination with a review of medical records were used to collect socio-demographic, risk factors and clinical characteristics of HIV positive individuals. Information collected include age, sex, hospitalization in the past 6 months, hospital visit in the past 12 months, under five children staying in daycare centers, homeless (current or past), prison (current or past), surgery in the past 1 year, household member’s hospitalization in the past 1 year, presence of percutaneous device in the past 1 year, oral antibiotic usage in the past 3 months, current use of Co-trimoxazole, most recent CD4 count.

### Specimen collection, transportation, culturing and bacterial isolation

Nasal and throat swabs were collected using sterile cotton swabs pre-moistened with sterile normal saline using the standard procedure (D’Avila et al. 2008; Kumar et al. 2013; Dhuria et al. 2013). Nasal specimens were obtained by rotating a single swab 2–3 times around the inside of the anterior nares, whereas throat specimens were obtained by swabbing the posterior pharynx and lateral walls of the pharynx (tonsillar area), without touching the buccal mucosa or tongue. Swabs were then inoculated into the Stuart’s Transport media and transported to the Ayder referral hospital medical microbiology laboratory within 3–4 h. Swabs were inoculated onto Mannitol Salt Agar (MSA) (Oxiod, Hampshire, UK) and incubated at 37 °C aerobically for 24 h. After incubation, yellowish colonies from the MSA plate were sub-cultured to Nutrient Agar (Oxiod, Hampshire, UK) for further biochemical characterization. Bacterial identification was done based on colony morphology, color of the colonies and the tube coagulase test. MRSA isolates were identified using Cefoxitin disc by the Kirby-Bauer disk diffusion method (CLSI 2013).

### Antimicrobial susceptibility pattern of MRSA to other antibiotics

The antimicrobial susceptibility testing of MRSA was detected using the modified Kirby-Bauer disk diffusion method according to the clinical laboratory standard institute (CLSI) guidelines (CLSI 2013). The following antimicrobials were used in their respective concentration: Ciprofloxacin (5 μg), Trimethoprim-Sulfamethoxazole (1.25/23.75 μg), Erythromycin (15 μg), Clindamycin (2 μg), and Amikacin (30 μg) (Hi-Media, India). These antimicrobials were selected based on the local usage to treat MRSA and considering the recommended antimicrobial agents for MRSA infections.

### Data processing and analysis

Variables from the demographic and clinical data obtained from the questionnaire and laboratory data were cleaned and entered into a computer. Statistical analysis was done using SPSS version 20.0 for windows. X^2^ (Chi square) test was used to determine the association of risk factors. P < 0.05, considered as statistically significant and multivariate analysis was used to differentiate the independent risk factors for *S. aureus* colonization.

### Quality control

Standard operating procedures (SOPs) were followed during sample collection, transportation, and processing steps. The quality of the culture media and antimicrobial disks were thoroughly checked using standard American Type Culture Collection (ATCC) reference strain of *S. aureus* ATCC 25923 obtained from Ethiopian Health and Nutritional Research Institute (EHNRI).

### Ethical clearance

This study was ethically approved by the Ethical Review Committee (Ref. No-ERC0453/2014), College of Health Science Mekelle University. Written consent was obtained from the study participants or guardians. Patients who were positive for MRSA were contacted by the respective doctors for further management. All information of participants were kept confidential.

## Results

A total of 249 HIV positive individuals attending HIV care service in four health facilities were included in the study. The majority of the participants were females 174 (69.9 %). The age range of participants were 5–72 years with a mean age of 35 years. The majority 103 (41.4 %) of the participants were in the age group of 30–39 (Table 1). The overall rate of *S. aureus* and MRSA colonization among the 249 study participants were 81 (32.5 %) and 6 (2.4 %) respectively. Distribution of MRSA by nasal, throat and both sites were 3, 2, 1 respectively and for S*. aureus* was 41 (50.6 %), 28 (34.6 %) and 12 (14.8 %) respectively.

**Table 1:** Analysis of risk factors for Nasal and Throat colonization of *S. aureus* in HIV positive individuals attending HIV care service in Northern Ethiopia, September 2014–February 2015

Variables	Frequency (%)	*S. aureus* colonization		
		No. (%)	OR (95 % CI)	P value
Age in years				
1–9^R^	10 (4.0)	1 (10)	NC	0.59
10–19	19 (7.6)	8 (42.1)		
20–29	35 (14.1)	10 (28.6)		
30–39	103 (41.4)	36 (35.0)		
40–49	55 (22.1)	18 (32.7)		
50–59	17 (6.8)	5 (29.4)		
60–69	7 (2.8)	1 (14.3)		
70–79	3 (1.2)	2 (66.7)		
Sex				
Female^R^	174 (69.9)	62 (35.6)	0.613 (0.334–1.123)	0.113
Male	75 (30.1)	19 (25.3)		
Hospitalization in the past 6 months				
No^R^	223 (89.6)	60 (26.9)	11.41 (4.12–31.62)	0.000
Yes	26 (10.4)	21 (80.8)		
Hospital visit in the past 12 months				
No^R^	61 (24.5)	28 (45.9)	0.463 (0.255–0.839)	0.011
Yes	188 (75.5)	53 (28.2)		
Household member’s hospitalization in the past 1 year				
No^R^	224 (90)	60 (26.8)	14.35 (4.732–43.516)	0.000
Yes	25 (10)	21 (84.0)		
Presence of percutaneous device in the past 1 year				
No^R^	189 (75.9)	33 (17.5)	18.91 (9.061–39.46)	0.000
Yes	60 (24.1)	48 (80.0)		
Oral antibiotic usage in the past 3 months				
No^R^	181 (72.7)	50 (27.6)	2.195 (1.232–3.912)	0.08
Yes	68 (27.3)	31 (45.6)		
Current use of trimethoprim-sulfamethoxazole				
No^R^	161 (64.7)	24 (27.3)	1.462 (0.827–2.583)	0.192
Yes	88 (35.3)	57 (35.4)		
Most recent CD4 count				
<200^R^	37 (14.9)	21 (56.8)	1.00	0.004
200–500	109 (43.8)	33 (30.3)	0.331 (0.153–0.713)	0.005
>500	103 (41.4)	27 (26.2)	0.271 (0.123–0.593)	0.001

The percent (%) of *S. aureus* colonization is the proportion of each category

*R* reference category

### Analysis of risk factors for *S. aureus* and MRSA colonization

Variables assessed as risk factors and found with a frequency of less than 10 % of the total 249 participants were excluded from the statistical analysis of risk factors for *S. aureus*. These include child aged under 5 years (0), homeless (4), prisoners (2), surgery in the past 1 year (3), recent intravenous antibiotic usage (0) and HIV positive individuals who have not started antiretroviral therapy (Pre-ART) (7). Two of the homeless, one of these previously prisoners, and two of these Pre-ART HIV patients were colonized with *S. aureus*. MRSA was not isolated from the prisoners, those with surgery in the past one year, and pre-ART, HIV patients, whereas one person among the homeless participants was colonized with MRSA.

Individuals in the age group of 70–79 years were more colonized by *S. aureus;* however, there was no statistical significance (p = 0. 59) (Table 1). All the six MRSA was isolated from the age groups of 20–49 years (Table 2). *S. aureus* colonization rate was higher among females 62 (35.6 %) as compared to males 19 (25.3 %). However, this was not statistically significant (p = 0. 113) (Table 1). All MRSA isolates 6 (3.4 %) were from females (Table 2).

**Table 2:** Analysis of risk factors for nasal and throat colonization of MRSA in HIV positive individuals attending HIV care service in Northern Ethiopia, September 2014–February 2015

Variables	Frequency (%)	MRSA colonization
		No. (%)
Age in years		
1–9^R^	10 (4.0)	0 (0)
10–19	19 (7.6)	0 (0)
20–29	35 (14.1)	2 (5.7)
30–39	103 (41.4)	3 (2.9)
40–49	55 (22.1)	1 (1.8)
50–59	17 (6.8)	0 (0)
60–69	7 (2.8)	0 (0)
70–79	3 (1.2)	0 (0)
Sex		
Female	174 (69.9)	6 (3.4)
Male	75 (30.1)	0 (0)
Hospitalization in the past 6 months		
No	223 (89.6)	4 (1.8)
Yes	26 (10.4)	2 (7.7)
Hospital visit in the past 12 months		
No	61 (24.5)	2 (3.3)
Yes	188 (75.5)	4 (2.1)
Household member hospitalization in the past 1 year		
No	224 (90)	4 (1.8)
Yes	25 (10)	2 (8.0)
Presence of percutaneous device in the past 1 year		
No	189 (75.9)	2 (1.1)
Yes	60 (24.1)	4 (6.7)
Oral antibiotic usage in the past 3 months		
No	181 (72.7)	3 (1.7)
Yes	68 (27.3)	3 (4.4)
Current use of trimethoprim-sulfamethoxazole		
No	161 (64.7)	2 (2.3)
Yes	88 (35.3)	4 (2.5)
Most recent CD4 count		
<200	37 (14.9)	3 (8.1)
200–500	109 (43.8)	1 (0.9)
>500	103 (41.4)	2 (1.9)

The percent (%) of MRSA colonization is the proportion of each category

*R* reference category

*Staphylococcus aureus* colonization rate was statistically significant in individuals with the history of hospitalization, percutaneous device usage within the past 1 year, patients with a household member’s hospitalization within past 12 months and patients with CD4 count lower than 200 cells/mm^3^ (p < 0.05) (Table 1). Similarly, MRSA colonization was higher in patients with a history of hospitalization (Table 2). Risk factors for the colonization of *S. aureus* with statistical significance in the bivariate analysis were further analyzed using multivariate analysis and were found to be significantly associated (Table 3).

**Table 3:** Multivariate analysis of independent risk factors associated with *S. aureus* colonization among HIV positive individuals attending HIV care service in Northern Ethiopia, September 2014–February 2015

Risk factors	AOR (95 % CI)	P value
Hospitalization in the past 6 months	9.97 (2.69–36.87)	0.001
Hospital visit in the past 12 months	0.31 (0.14–0.697)	0.004
Presence of percutaneous device in the past 1 year	20.695 (8.75–48.96)	0.000
Family member’s hospitalization in the past 1 year	12.97 (3.48–48.30)	0.000
Most recent CD_4_ count		
<200		0.016
200–500	0.33 (0.12–0.91)	0.033
>500	0.22 (0.08–0.06)	0.004

*AOR* adjusted odds ratio

### Antimicrobial susceptibility pattern of MRSA isolates

Antimicrobial susceptibility testing was performed on the six MRSA isolates against the commonly used antibiotics. Accordingly, 16.7, 33.3 and 50 % resistance were observed against Ciprofloxacin, Trimethoprim-Sulphamethaxazole, Clindamycin and Erythromycin respectively. Interestingly, all the isolates have shown 100 % sensitivity to Amikacin. However, three of the MRSA isolates (50 %) have shown multidrug resistance and all of them were isolated from HIV patients in hospital.

## Discussion

Our result indicates that the prevalence of *S. aureus* 81 (32.5 %) and MRSA 6 (2.4 %) was lower than similar studies conducted in San Francisco 55.2 % for *S. aureus* and 27.6 % for MRSA (Miller et al. 2003) and India 50.7–81 % for *S. aureus* and 18.3–42 % for MRSA (Dhuria et al. 2013). MRSA prevalence of 2.4 % was similar to the 5.1 % reported from Singapore (Kyaw et al. 2012). However, it was lower than report from other African nations with a rate of 20 % in Nigeria (Edem et al. 2013) and 21 % in Kwazu Natal Hospital South Africa (Heysell et al. 2011).

The low prevalence of *S. aureus* and MRSA colonization in our study might be due to the rare visit of these HIV patients to the health facilities, since repeated visits to health care centers, or contact with hands of health care workers in HIV infected individuals is the major risk factor for the colonization (Hidron et al. 2010; Lowy 1998; Government of Western Australia Department of Health 2013).

In our study, the inclusion of throat swabs increased the sensitivity of nasal screening of *S. aureus* and MRSA by 34.6 and 33.3 %, respectively, which was also shown by other studies conducted in Bristol, UK (Chow et al. 2012), Singapore (Kyaw et al. 2012) and Switzerland (Mertz et al. 2007).

In this study, recent hospitalization was associated with *S. aureus* colonization. This was also showed by other studies conducted in Singapore (Kyaw et al. 2012; Villacian et al. 2004) and TX, USA (Onorato et al. 1999). *S. aureus* and MRSA colonization rate was higher among those who visited hospitals, in the last twelve months; this is in line with the result of other study conducted in Singapore (Kyaw et al. 2012). Presence of percutaneous device within the past year was found to have a strong association with *S. aureus* and MRSA colonization. This result was supported by other studies conducted in Singapore (Kyaw et al. 2012) and in TX, USA (Onorato et al. 1999).

MRSA colonization was higher in those with Low CD4 count (less than 200/mm^3^) which was also shown by other studies conducted in Singapore (Villacian et al. 2004) and in Dallas, TX, USA (Cenizal et al. 2008). In our study the antimicrobial susceptibility pattern of MRSA isolates showed 100 % sensitivity to Amikacin which was similar to the study in India (Dhuria et al. 2013).

## Conclusions

In this study there was a lower prevalence of MRSA; however, high degree of *S. aureus* colonization among the HIV patients. History of hospitalization, percutaneous device usage, patients with a household member’s hospitalization and CD4 count lower than 200 cells/mm^3^ were significantly associated with *S.aureus* colonization. MRSA isolates from HIV patients in hospital were more resistant than MRSA isolates obtained from health centers. All MRSA isolates were 100 % sensitive to Amikacin.
